# Using a Pulse Protocol to Fix the Individual Dosage of Transcranial and Transspinal Direct Current Electrical Stimulation

**DOI:** 10.3390/life13061376

**Published:** 2023-06-12

**Authors:** Evgeny Blagovechtchenski, Svetlana Kostromina, Alla Shaboltas

**Affiliations:** Laboratory of Behavioural Neurodynamics, St. Petersburg State University, St. Petersburg 199034, Russia

**Keywords:** noninvasive brain stimulation, tDCS, transspinal stimulation, motor control, locomotion, stimulation protocols

## Abstract

The non-invasive current stimulation protocol differs significantly between the brain and spinal cord, such that when comparing the two, there is a clear predominance of protocols using transcranial direct current stimulation (tDCS) for the brain and of protocols using pulsed stimulation for the spinal cord (psSC). These protocols differ in their effects on the central nervous system and in such important parameters as stimulation intensity. In most cases, tDCS has a fixed amplitude for all subjects/patients, while psSC is usually chosen on a case-by-case basis, according to the thresholds of muscle responses. In our opinion, it is possible to use the experience of identifying thresholds during psSC to adjust the dose of the direct current for transcranial and transspinal electrical stimulation, an approach that may provide more homogeneous tDCS data.

Of particular interest herein, non-invasive brain stimulation is a widely used protocol that involves stimulation with a direct current, leading to long-lasting neuroplastic changes [1,2]. Active use of such stimulation is beginning to increase in clinics, as its advantages include simplicity and a low cost [3,4].

## 1. Major Targets in the Central Nervous System

When the central nervous system is the target of such an impact, the direct targets include the cerebral cortex, cerebellum, and spinal cord, due to the anatomical proximity of these structures to the surface [3,5]. However, they have pronounced structural differences, including a different laminar structure of the organization of neuronal layers, different structures of afferents and efferents, and different tissue configurations between the structure and the surface [3,6,7]. However, in relation to the anatomical features, the question arises of how to select the intensity of the impact on the desired structure.

## 2. The Problem of Selecting the Direct Current Stimulation Intensity

Most researchers adopt a simple approach: “use the literature data” and set a fixed intensity, but this has its pros and cons. A great convenience is an ability to compare collected data with the literature data, thus making it possible, for example, to draw conclusions about the effect of transcranial electrical stimulation with an intensity of, for example, 1.5 mA on different areas of the brain and in different tasks [8,9,10]. However, there are increasingly conflicting results in the literature, and some meta-analyses even show ceiling effects or null results, possibly due to the large interindividual variability up to contradirectionality [11,12].

It is worth emphasizing the role of studies in the impact on the motor cortex. When stimulating the primary motor cortex, we can objectively assess the effect of stimulation using electromyography of the target muscles, which makes it possible to judge the nature of the stimulation effect [13]. Such studies have led to the use of “activating” and “inhibitory stimulation” (increase or decrease in motor-evoked potentials (MEPs)), which began to be automatically transferred effects to other areas of the brain, including all stimulation parameters [14]. Evidence in the literature shows that subjective evaluation of stimulation effects can lead to incorrect dosage parameters [15].

The question is the translation of transcranial direct current stimulation (tDCS) protocols from healthy people to patients. It is not trivial since the individual patient’s medical history adds to the variability in effects. It complicates introducing tDCS protocols into the clinic since one cannot see the effects.

A separate issue is how to dose stimulation in children of different ages. Now, as far as we know, there is no solution to this issue.

## 3. Variability in tDCS Results

If we consider the problem retrospectively, all the data initially appear relatively homogeneous: anodic stimulation is “activated” and the cathodes “inhibit” one or more areas of the cerebral cortex [16]. Such data were obtained based on the MEP assessment from transcranial magnetic stimulation (TMS) after the corresponding tDCS of the primary motor cortex, which was included in the textbooks [17]. However, over time, evidence has begun to accumulate that even small changes in stimulation intensity can significantly affect the results. For example, a change of 0.5–1 mA can negate the effects, whereas those obtained during stimulation of the primary motor cortex at an intensity of 1.5 mA are nullified at an intensity of 2–2.5 mA [18,19]. Some studies also showed an inversion of the effects when the stimulation intensity changed from inhibition to activation [20]. These linear effects are not observed here, and this generally calls into question the definition of the effects of “anodic” and “cathodic” stimulation [21,22]. It should also be noted that similar effects were observed with transspinal direct current stimulation (tsDCS) [6].

In our opinion, the problem lies in the multidirectional effects of stimulation at different intensities and intragroup variabilities. Often, at the same stimulation intensity, different subjects have multidirectional responses, and an increase in the sample size can lead to the “ceiling” effect, where, even with pronounced stimulation of a significant part of the group of subjects, on average, we do not observe effects due to the multidirectional action of the other part of the group [3,11].

Quite a lot of research confirms the fact that the effect of tDCS on MEPs in humans is subject to high individual variability [13,23,24,25]. Based on the analysis of the results of the study of the effects of non-invasive brain stimulation (NIBS) on cognitive and motor skills, we can conclude that the results are inconsistent because the effectiveness of stimulation depends on many factors (gender, age, initial cognitive and motor data of patients).

An important factor influencing the effectiveness of NIBS is high individual variability; the solution may be to tailor stimulation dosage and electrode placement to the individual brain model, which one can achieve using computer simulations, but this approach is more costly and time-consuming.

The large intra- and inter-individual variability shown by several studies conducted on a large sample of healthy subjects may make it difficult to see task-specific effects [26].

This variability is based on several factors, many of which are constant, such as age, sex, and genetic polymorphism. Therefore, it is important to control them with a straightforward experimental design [27]. Another critical factor that is difficult to control is ongoing cortical activity and subject attention. For example, the aftereffects of TMS increase if the subject focuses on the stimulated hand, while the effects decrease if the subject directs attention to the unstimulated hand [28]. The gender of the subject/patient is also of great importance [29]. The menstrual cycle can affect the excitability and plasticity of the cortex; for example, the effects of TMS have a more significant impact on the subject at cycle day 14, as estradiol enhances synaptic potentiation by acting on voltage-gated sodium channels [30]. Another potential source of variation is genetic factors. As an example: subjects with a polymorphism in the Val66Met gene encoding the brain-derived neurotrophic factor (BDNF), which affects cognitive functions, have a reduced sensitivity to HCM protocols [31].

Due to the heterogeneity of the patient population and the variety of protocols used in studies, it is difficult to conduct a systematic review and quantify the therapeutic benefit of different regimens of transcranial electrical stimulation. Most trials are not double-blind, and the evidence for their efficacy and safety is unknown [32].

In our opinion, one of the main reasons for this phenomenon may be individual differences in sensitivity to stimulation intensity and the need for the personalized selection of stimulation parameters. Conversely, many researchers prefer small samples with statistical significance in the findings, which may not occur when the sample size is expanded.

However, if we compare the protocols for stimulation between direct current and pulsed current, we find quite interesting points of divergence that might allow for a more personalized approach.

## 4. Differences in Protocols

The results of comparing the protocols used at different levels are of great interest. [3,5,33]. Thus, protocols using an impulse structure are rarely used to affect the cerebral cortex, which is a common approach for the spinal cord, likely because the mechanisms of stimulation have different purposes. In the case of tDCS, the main goal is to shift the membrane potential of a large pool of cells in one direction or another, which can affect the planning/execution/control of a particular motor program [4,34]. It is worth emphasizing that the mechanisms of the effect of tDCS on the brain still need to be better understood. There are several different hypotheses about where and how the shift in membrane potential occurs and in which cells it occurs [7]. A significant difficulty in assessing the effect of tDCS on the cerebral cortex or cerebellum is the complex anatomical structure—cerebral gyrus and sulci [8,35]. It remains a debatable question: where exactly does the most significant effect of current on neurons occur [7,36]? This problem may be because the effect of tDCS does not lead to the production of action potentials, and we can only indirectly judge the results. And since such effects can be affected by a particular person’s anatomy and physiology features, the conclusions’ reliability decreases. In the case of psSC, the main goal is the direct or indirect activation of motor neurons, where mediated activation can occur through the impulse activation of afferents or the involvement of locomotor pacemakers [5,33]. Therefore, with psSC, it becomes possible to determine the moment at which the acting potentials of motor neurons were generated and, accordingly, to measure the thresholds of such activation. There are significant differences in the use of such protocols, which are especially relevant to clinics.

## 5. Existing Solutions for Finding Thresholds

According to the available literature data, there are currently several proposals on how to transition to individual tDCS and tsDCS parameters. The simplest approach, at first glance, is to use the impulse protocol for the primary motor cortex [37,38]. In TMS experiments, the classic approach is to find thresholds for first dorsal interosseous (FDI) or abductor pollicis brevis (APB) muscles and use them to stimulate other target cortex areas, assuming that similar thresholds can be applied to the selected areas [39,40]. However, this approach is neither entirely effective nor practical for non-invasive electrical stimulation. First, electrical stimulation of the primary motor cortex cannot be as localized as TMS, which leads to a significant increase in stimulation intensity. Second, increasing the current strength leads to increased pain in the subject/patient. In the literature, few results of such stimulations are provided, where effects are observed starting from an intensity of 58 mA. We consider that such studies are unique and cannot be considered a common approach.

Another logical solution could be to use MEPs from TMS in the cases of tDCS and tsDCS as well, which will allow the objective assessment of the excitability of the corticospinal system and thus the creation of a relative scale for tDCS [19,41]. A disadvantage of this method is the presence of a TMS setup. In this case, there is one significant limitation: not all laboratories/clinics can afford the cost of a TMS unit, which is many times greater than the cost of tDCS and tsDCS. This is especially true in terms of the widespread use of such an approach in clinics.

Other interesting approaches to tDCS dosing have also been suggested, including E-field modeling based on individual MRI [37,42]. However, in our opinion, everything can be reduced to the use of more expensive equipment, which negates the advantages of using tDCS, a cheap, simple, and relatively effective method of affecting the nervous system.

## 6. Possible Solutions

We propose using impulse stimulation of the spinal cord, as well as determining the thresholds of motor responses as a relative parameter for dosing tDCS and tsDCS. We consider that it is possible to create a transparent scale to translate the MEP threshold finding in the case of psSC to the individual tDCS and tsDCS scale; this approach requires additional research, but it considers such important parameters as the excitability of the nervous system, the average skin resistance, and others that are difficult to deliberate comprehensively without trialing electrical stimulation.

This issue is significant for applying the tDCS method in the clinic. It is crucial for patients with specific body systems disorders, which does not allow direct translation of the effects of tDCS obtained on healthy people. As mentioned above, a significant issue in the dosage of tDCS is using this method for children of different ages.

Of course, it is difficult to translate the effects of psSC into the tDCS protocol. The effects of tDCS are polysynaptic, while psSC is an analog of H-reflex and has an oligosynaptic nature. However, the effects of both protocols reflect the underlying excitability of the nervous system. Therefore H-reflex is used in a person’s most superficial neurological examination. In our opinion, the correction of tDCS, considering the general excitability of the nervous system, can significantly enhance the effects of such stimulation. In addition, the use of psSC instead of TMS (as a probe for the excitability of the corticospinal system) significantly reduces the cost of the equipment. Additonally, using TMS requires a higher personnel qualification, which is important for implementation in the clinic.

Creating such a transparent scale is a complex and unambiguous solution. It is currently impossible to directly compare the amplitude of MEP and the intensity of transcranial stimulation. The roughest scales could be used to begin with. For example, linearly compare the intensity of 1.5–3.0 mA common for transcranial stimulation with 20–150 mA used to assess the MEP of the distal muscles of the arms during pulsed stimulation of the cervical thickening to find thresholds. Finding thresholds for distal muscle activation (eg, FDI or APB) is not a painful procedure, unlike the assessment of the recruitment curve, and can be an objective assessment of individual sensitivity to non-invasive brain stimulation. Moreover, this method of stimulation is currently widely practiced in the clinic. This approach should imply the use of the same equipment for identifying thresholds, but most tDCS stimulators do not allow the delivery of pulsed currents in the range of 30–200 mA, which is necessary for assessing excitability thresholds for psSC. This again highlights the different communities; for example, those who use pulsed stimulation use a different stimulator.

In our opinion, if researchers using tDCS in their studies also can use the psSC protocol to find the threshold of at least the FDI muscle, then it should be used as an additional criterion. It will make it possible to assess the individual assessment of excitability of the nervous system, which will make it possible to more correctly assess the effects of tDCS and correctly interpret the data obtained. Using the psSC protocol to find the threshold takes about 10 min, including placing an electrode on the FDI muscle. The combination of psSC and tDCS protocols can significantly improve the quality of research in non-invasive stimulation and its implementation in the clinic.

## Data Availability

Not applicable.
